# Evaluation of the performance of both machine learning models using PET and CT radiomics for predicting recurrence following lung stereotactic body radiation therapy: A single‐institutional study

**DOI:** 10.1002/acm2.14322

**Published:** 2024-03-04

**Authors:** Hikaru Nemoto, Masahide Saito, Yoko Satoh, Takafumi Komiyama, Kan Marino, Shinichi Aoki, Hidekazu Suzuki, Naoki Sano, Hotaka Nonaka, Hiroaki Watanabe, Satoshi Funayama, Hiroshi Onishi

**Affiliations:** ^1^ Department of Advanced Biomedical Imaging University of Yamanashi Chuo Yamanashi Japan; ^2^ Department of Radiology University of Yamanashi Chuo Yamanashi Japan; ^3^ Imaging Center Fujita Medical Innovation Center Tokyo Tokyo Japan; ^4^ Department of Radiology Fuji City General Hospital Fuji Shizuoka Japan; ^5^ Department of Radiology Yamanashi Central Hospital Kofu Yamanashi Japan; ^6^ Department of Radiology Hamamatsu University school of medicine Hamamatsu Shizuoka Japan

**Keywords:** lung cancer, machine learning, PET imaging, radiomics, SBRT

## Abstract

**Purpose:**

Predicting recurrence following stereotactic body radiotherapy (SBRT) for non‐small cell lung cancer provides important information for the feasibility of the individualized radiotherapy and allows to select the appropriate treatment strategy based on the risk of recurrence. In this study, we evaluated the performance of both machine learning models using positron emission tomography (PET) and computed tomography (CT) radiomic features for predicting recurrence after SBRT.

**Methods:**

Planning CT and PET images of 82 non‐small cell lung cancer patients who performed SBRT at our hospital were used. First, tumors were delineated on each CT and PET of each patient, and 111 unique radiomic features were extracted, respectively. Next, the 10 features were selected using three different feature selection algorithms, respectively. Recurrence prediction models based on the selected features and four different machine learning algorithms were developed, respectively. Finally, we compared the predictive performance of each model for each recurrence pattern using the mean area under the curve (AUC) calculated following the 0.632+ bootstrap method.

**Results:**

The highest performance for local recurrence, regional lymph node metastasis, and distant metastasis were observed in models using Support vector machine with PET features (mean AUC = 0.646), Naive Bayes with PET features (mean AUC = 0.611), and Support vector machine with CT features (mean AUC = 0.645), respectively.

**Conclusions:**

We comprehensively evaluated the performance of prediction model developed for recurrence following SBRT. The model in this study would provide information to predict the recurrence pattern and assist in making treatment strategies.

## 1 INTRODUCTION

Lung cancer is the leading cause of cancer‐related death, and 5 year‐survival rates for non‐small cell lung cancer (NSCLC), which accounts for 80%−85% of all lung cancers, remain extremely low.^1^, ^2^, ^3^, ^4^ In a review of 10 articles from 1988−1998 on radical radiation therapy for stage I NSCLC, Sibley et al. reported that distant metastatic death occurred in 30% of the patients analyzed and local recurrence in 30%.^5^, ^6^ These shows that metastasis is a critical factor in the survival of NSCLC patients. Recently, target delineation, respiratory motion management, conformal treatment planning, and image‐guided radiotherapy (IGRT) for each fraction have been performed with high precision, making stereotactic body radiation therapy (SBRT) feasible. SBRT can deliver conformal high‐dose radiation to the target accurately within a few fractions. Previous reports have shown high local control rates (85%−90%) comparable to surgery in treatment with SBRT.^7^ The guidelines for the treatment of lung cancer recommend treatment with SBRT for patients who are medically ineligible for surgery or who refuse surgery.^8^ However, the retrospective multi‐institution study to review patients who were treated by hypofractionated stereotactic radiotherapy for their stage I NSCLC showed that local, regional nodal, and distant recurrence rates were 14.0%, 11.3%, and 19.8%, respectively.^9^ Therefore, it is imperative to predict the status of recurrence precisely and noninvasively prior to treatment to select the suitable treatment strategy for the patient's specific risk of recurrence.

Medical imaging is a minimally invasive method to acquire tumor characteristics. 2‐[18F]‐fluoro‐2‐deoxy‐D‐glucose (^18^F‐FDG) positron emission tomography (PET) with computed tomography (CT), which has both anatomical and functional information, has often been employed to stage patients diagnosed with NSCLC^10^. PET imaging methods are important for noninvasive staging. The performance of CT and PET/CT images has been compared in lymph node staging of lung cancer. In the previous report, the sensitivity and specificity of CT imaging were approximately 55% and 81%, respectively, whereas the sensitivity and specificity of PET imaging were approximately 62%−76.9% and 86%−89.1%, respectively for the diagnosis of mediastinal lymph node metastases in lung cancer.^11^, ^12^, ^13^ PET imaging has shown higher performance than CT imaging, however, qualitative diagnosis for medical imaging is subjective and has limited precision.

Radiomics is a novel approach for the comprehensive analysis of a large number of medical images, initially documented in 2014 by Lambin et al.^14^ Numerous image features can be derived by using high‐throughput radiomic analysis on medical images. Patterns of extracted image features have been reported to correlate with the gene phenotype of tumors, and the usefulness of radiomics in staging, prognosis, diagnosis, and prediction of treatment response has been reported.^15^, ^16^, ^17^, ^18^, ^19^ Radiomic features derived from PET/CT imaging have been reported to have the performance to predict prognosis and treatment response in NSCLC patients who have undergone radiotherapy.^20^, ^21^, ^22^, ^23^, ^24^, ^25^ Similarly, previous studies have reported that radiomic features derived from PET, CT, or MR images may be useful in predicting recurrence.^26^, ^27^, ^28^ Recently, Onozato et al. demonstrated the potential of radiomic features extracted from PET/CT images in assessing the risk of local recurrence^29^; Lucia et al. showed that radiomic features extracted from PET and CT images may be useful in predicting regional and distant recurrence.^30^ However, while these studies have reported on the performance of models that predict specific types of recurrence using radiomic features extracted from specific modalities, comprehensive data on performance in predicting local, regional, and distant recurrence are lacking. To our knowledge, no radiomics study has evaluated the predictive performance of both machine learning algorithms using PET radiomic features and CT radiomic features to predict local, regional, and distant recurrence patterns, respectively.

The purpose of this study was to evaluate the performance of machine learning models predicting local, regional, and distant recurrences of patients diagnosed with NSCLC. Briefly, radiomic features were extracted from PET and CT images acquired prior to the SBRT, and various feature selection algorithms and machine learning algorithms were used to develop models to predict post‐treatment recurrence in NSCLC patients, and we identified the models with high prediction performance by comprehensive evaluation.

## 2 MATERIAL AND METHOD

### 2.1 Patient characteristics

The medical records of 82 patients diagnosed with NSCLC who were treated with SBRT between January 2007 and December 2015 and performed PET/CT imaging prior to SBRT were reviewed. The details of tumor characteristics were adenocarcinoma, squamous cell carcinoma, large cell carcinoma, NSCLC non‐specified, and not biopsy proven. The inclusion criteria were as follows: (1) diagnosis of primary NSCLC, (2) clinical stage I (T1a‐T1bN0M0 and T2aN0M0) based on TNM classification (seventh edition).^31^ (3) availability of detailed patient information. Furthermore, patients with synchronous multiple lung cancers and those without remaining patient clinical characteristics: sex, age, pathology, Tstage, cancer location, treatments prescription dose, follow‐up period, and recurrence and metastases information  were excluded. For each patient in this study, the prescribed dose ranged between the biologically effective dose with α/β = 10 (BED_10_) of 96 and 134.4 Gy at 95% of the target volume receiving the prescribed dose. The range of BED_10_ values for the prescribed doses in this study was narrower than the range in the previous study.^32^

Diagnosis of recurrence was based on CT, PET/CT, or MRI findings, while histological confirmation of recurrence was not mandatory. Recurrences were confirmed pathologically in 4.9%‐local recurrence, 2.4%‐ regional lymph node metastases, 4.9%‐distant metastases of patients, and confirmed via CT, PET/CT, or MRI in 95.1%‐local recurrence, 97.6%‐regional lymph node metastases, 95.1%‐distant metastases in patients. The median follow‐up duration was 38 months.

### 2.2 PET/CT imaging

Patients were performed PET/CT imaging at Kofu Neurosurgical Hospital, PET center. The PET/CT images were acquired using Biograph Duo LSO (Siemens Healthineers, Knoxville TN, USA) without time of flight capability. All patients were fasted for 6 h. At 1 h after ^18^F‐FDG injection (3 MBq/kg), a CT scan (110 kV; 44−87 mA; slice thickness 5.0 mm; and transverse field of view 50 cm) was performed for attenuation correction, followed by a PET scan from the neck to the thigh. Visualization of radioactive tracers was performed in 3D mode (2 min per bed position). PET images were reconstructed using CT for attenuation correction with the ordered subsets expectation maximization algorithm (8 subsets, 2 iterations) with a voxel size of 4 × 4 × 2 mm.

### 2.3 RT method

Patients were treated with SBRT using EXL‐15DP (Mitsubishi Electric, Japan) coupled to a CT scanner Hi‐SpeedDX (GE Yokogawa Medical Systems, Japan), both of which shared a common couth, and Elekta Synergy unit (Elekta AB, Stockholm, Sweden)  coupled to a CT scanner Aquilion LB (Canon Medical Systems Corporation, Japan), both of which shared a common couth at the University of Yamanashi hospital. The Synergy unit were equipped with an Agility gantry head, which has 160 MLC leaves of 5 mm. IGRT with on‐rail CT system installed in the treatment room was performed for each treatment fraction. Respiratory motion management was performed, in principle during inhalation, a breath‐holding technique using Abches, which is a respiratory indicator used for respiratory motion management.^33^, ^34^ The prescription doses and fractionations were as follows: 48 Gy/ 4 Fr, 50 Gy / 4 Fr, or 55 Gy / 4 Fr for T1, and 60 Gy / 10 Fr or 70 Gy / 10 Fr for T2 was adopted as the dose in cases where a tumor was located close to an organ at risk. For all treatment plans, SBRT was performed using three‐dimensional conformal radiation therapy (3DCRT) or dynamic arcs. All planning CT images were acquired using Aquilion LB CT (Canon Medical Systems Corporation, Japan) and Hi‐Speed DX/I (GE Yokogawa Medical Systems, Japan) with the following settings: 120 kV, 250 mA, 500 ms, 125 mAs. CT images were reconstructed using FC13 kernel with a voxel size of 0.70 – 1.07 × 0.70 – 1.07 mm. The slice thickness of CT images was 1.2–3.0 mm.

### 2.4 Tumor delineation

Tumors were delineated for each of the CT images used for treatment planning and the pre‐treatment PET images. The expert radiation oncologists delineated the primary tumors (GTV) on the CT images, which were used for treatment planning. The tumors regions of interest on the PET images were semi‐automatically segmented using the PET‐Tumor‐Segmentation tool for PET images implemented in the 3D‐Slicer software (Version 4.11.20210226, www.slicer.org/). This method is Graph‐based Segmentation and generates tumor or lymph node segmentations with one click. Previous studies described this algorithm in detail.^35^

### 2.5 Radiomic feature extraction

A total of 111 unique quantitative radiomic features were extracted from both the PET images and the treatment planning CT images using Pyradiomics (version 3.0.1), an open‐source software module.^36^ Radiomic features included the shape features (e.g., volume, sphericity, and compactness), the first order statistics features (e.g., maximum, mean, standard deviation, and entropy), and the texture features (e.g., gray level co‐occurrence matrix, gray level run length matrix, and neighborhood gray tone difference matrix). The details of all 111 radiomic features used in this study were summarized in the supplementary document Table A. The extracted features were normalized by min‐max scaling to reduce the influence of scale differences for each feature.

### 2.6 Statistical analysis and machine learning methods

**FIGURE 1:**
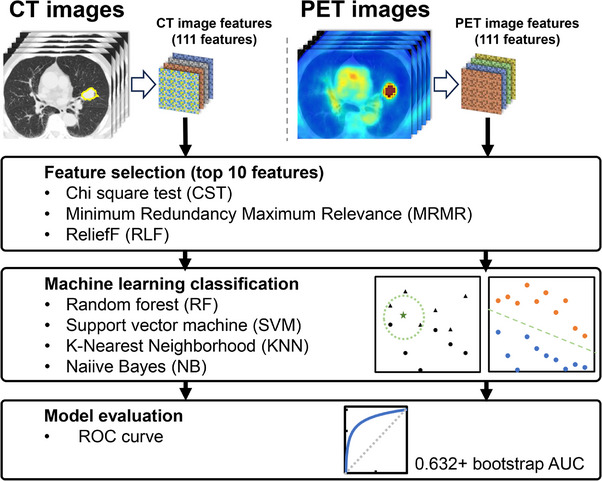
Workflow of prediction using radiomics and machine learning. Radiomic features of the CT image and the PET image were extracted from each CT image volume for treatment planning and from the PET image volume acquired prior to radiation treatment. Next, the useful features for predicting each outcome were selected. Then, the top 10 features for each of the three feature selection algorithms were used to create feature subsets for each endpoint. The four machine learning classification algorithms were then used to predict each endpoint. Finally, the performance of each model in predicting each endpoint was evaluated using the mean 0.632+ bootstrap area under the curve (AUC) method over 1000 iterations.

The endpoint of this study was recurrence status. Specifically, each recurrence pattern was categorized as follows: local recurrence (LR); regional lymph node metastases (RM); distant metastases (DM); LR or RM (LR—RM); LR or DM (LR—DM); RM or DM (RM—DM); and LR, RM, or DM (LR—RM—DM). Figure 1 shows the workflow of prediction using radiomics and machine learning. A feature selection procedure was performed to select useful features for predicting each endpoint. Therefore, we employed three filter‐based feature selection algorithms: the Chi‐square test (CST), the minimum redundancy maximum relevance (MRMR) algorithm, and the Relief algorithm (RLF) to reduce the radiomic feature dimensions using MATLAB R2022b (MathWorks, Natick, MA, USA), respectively. In this study, all feature selection algorithms; CST, MRMR, and RLF for classification available in MATLAB 2022b were used. The CST algorithm can select features that are important for predicting endpoints by testing whether each feature is independent of response using a chi‐squared test. The MRMR algorithm minimizes redundancy in the feature set and maximizes relevance to endpoints by quantifying the mutual information between features as well as between features and responses.^37^, ^38^ The RLF algorithm is a conventional feature selection method developed by Konnonenko et al.^39^ These algorithms have been used in predicting prognosis, and predictive models using them have been shown to have superior predictive accuracy.^40^, ^41^, ^42^, ^43^ Each selected radiomic features from CT and PET images were ranked based on the scores related to each endpoint, respectively. Based on each rank, 10 best‐ranked features were selected, and the feature subsets were created.^16^, ^44^, ^45^ In summary, we employed three different feature selection algorithms to pick out PET or CT radiomic features associated with each of the seven endpoints. As a result, a total of 42 feature subsets were generated.

We then developed binary classification models based on four different machine learning algorithms to predict each endpoint and compared predicting performance. Four machine learning algorithms, Random forest (RF), Support vector machine (SVM), k‐nearest neighbor (KNN), and Naïve Bayes (NB), were used to develop the prediction models. Therefore, a total of 168 prediction models were developed by using combinations of 42 feature subsets and four different machine learning algorithms. The area under the curve (AUC) calculated from receiver operating characteristic (ROC) curve analysis was used as the performance metric for each model in predicting each endpoint. To assess the generalization performance of the developed prediction model, the AUC was calculated using the 0.632+ bootstrap method in this study. The 0.632+ bootstrap method has demonstrated lower variance, bias, and mean squared error for a small number of samples and many features. Previous studies described the 0.632+ bootstrap AUC metric in detail.^46^, ^47^, ^48^ Following this method, we resampled the training and test datasets with 1000 iterations, splitting the data into approximately 52 (63.4%) and 30 (36.6%) cases, respectively. The hyperparameter of each machine learning algorithm was optimized to maximize the value of the mean AUC as shown in Table 1. Finally, to evaluate prediction performance, the mean AUCs were compared between the machine learning algorithms and between the predictive models based on PET and CT features. Multiple comparisons of predictive performance between machine learning algorithms were performed using Friedman's test. A paired t‐test was performed to compare the predictive performance between the models based on PET and CT features. These statistical analyses were performed using MATLAB R2022b (MathWorks, Natick, MA, USA). In this study, a one‐sided test was used to evaluate whether there was a significant difference in predictive performance between the models.

**TABLE 1:** Machine learning model and each hyperparameter settings to optimize prediction model.

Machine learning model	Hyperparameter settings
Random forest (RF)	“NumLearningCycle” was set to 100. “Learners” used in ensemble was the template tree.
Support vector machine (SVM)	The kernel function was set to “rbf,” “Gaussian,” “linear,” and “polynomial” and each kernel scale was auto. “BoxConstraint” was set to 0.01, 0.1, 0.50, 1, 2, 5, 10, 50, 100, 200, 500, and 1000.
k‐nearest neighbor (KNN)	“NumNeighbors” was set to 1, 5, 10, and 20. “Distance” metrics such as “euclidean,” “cosine,” “minkowski,” and “spearman” was used to find the distance between a dataset and a query point.
Naïve Bayes (NB)	The normal Gaussian, uniform, Epanechnikov, and Triangular distribution was used for the data distribution to model the data.

## 3 RESULTS

### 3.1 Patient characteristics

Table 2 shows the clinical characteristics and demographics of the patients. Within 38 months of Median follow‐up, LR was observed in 15 (18.29 %), RM in 19 (23.17 %), DM in 25 (30.48 %), LR‐RM in 27 (32.93 %), LR‐DM in 34 (41.46 %), RM‐DM in 33 (40.24 %), and LR‐RM‐DM in 39 (47.56 %), respectively.

**TABLE 2:** Patient characteristics.

Characteristic		Overall *n* = 82
Sex	Male	66 (81%)
	Female	16 (19%)
Age		79.2 ± 6.2
Pathology	Adenocarcinoma	35 (43 %)
	Squamous cell carcinoma	24 (29 %)
	Large cell carcinoma	1 (1%)
	NSCLC non‐specified	11 (13 %)
	Not biopsy proven	11 (13 %)
T stage	T1a	28 (34 %)
	T1b	10 (12 %)
	T2a	44 (54 %)
Cancer location	Peripheral	73 (89 %)
	Central	9 (11 %)
Treatments prescription dose	48‐56 Gy / 4 Fr	66 (80 %)
	60 Gy / 10 Fr	5 (6 %)
	70 Gy / 10 Fr	11 (13 %)
Follow‐up period		38 months (median)
		(Range: 6−132)
Recurrence and metastases	Local recurrence (LR)	15 (18.29 %)
	Regional lymph node metastases (RM)	19 (23.17 %)
	Distant metastases (DM)	25 (30.48 %)
	LR‐RM	27 (32.93 %)
	LR‐DM	34 (41.46 %)
	RM‐DM	33 (40.24 %)
	LR‐RM‐DM	39 (47.56 %)

### 3.2 Radiomic feature selection and ROC curve analysis

**FIGURE 2:**
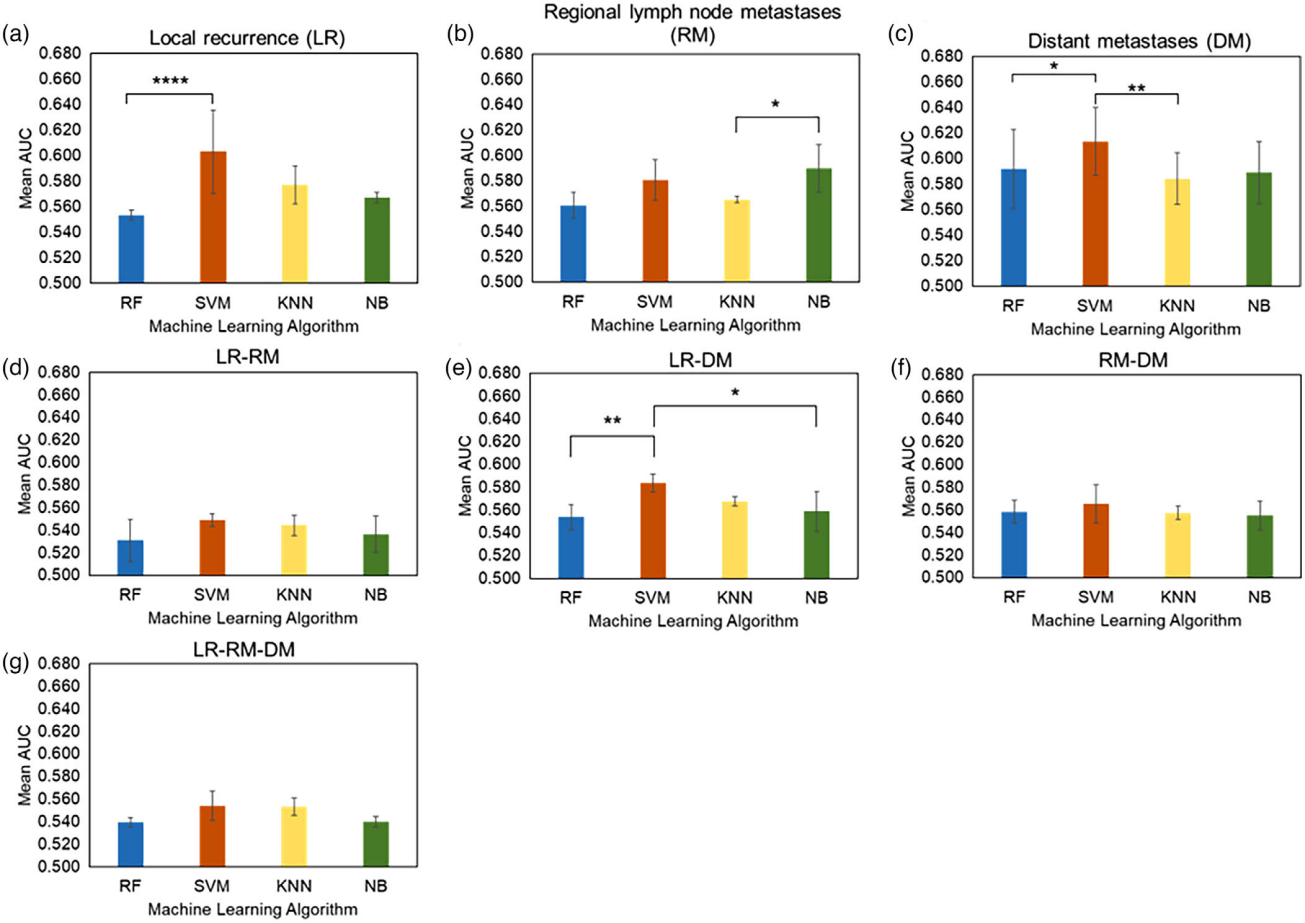
Mean AUC of all models using each machine learning algorithm in predicting each endpoint. The vertical axis shows the mean AUC of all models, and the horizontal axis shows the machine learning algorithms used for predicting each endpoint: (a) Local recurrence (LR), (b) Regional lymph node metastases (RM), (c) Distant metastases (DM), (d) LR‐RM, (e) LR‐DM, (f) RM‐DM, (g) LR‐RM‐DM. In each graph, blue, red, yellow, and green represent Random Forest (RF), Support vector machine (SVM), k‐nearest neighbor (KNN), and Naïve Bayes, respectively. Each error bar represents the standard deviation calculated from the AUC observed in each machine learning model. * *p* < 0.05; **p < 0.01; *** *p* < 0.005; **** *p* < 0.001.

The overall classification performance of the four machine learning models for each endpoint was compared by the mean AUC(Figure 2). The highest mean AUCs were observed for the models using SVM in predicting all endpoints except RM. The mean AUCs of the model using SVM in predicting LR, DM, LR‐RM, LR‐DM, RM‐DM, and LR‐RM‐DM were 0.603, 0.613, 0.549, 0.584, 0.566, and 0.554, respectively. Regarding LR prediction, the SVM models showed significantly higher predictive performance than the RF models (*p* < 0.001). For RM prediction, the NB models achieved the highest mean AUC of 0.590, showing significantly higher performance than the KNN models (*p* = 0.034). In predicting, DM, the SVM models significantly outperformed both the RF models and KNN models (*p* = 0.037, *p *< 0.01, respectively). Similarly, for LR‐DM prediction, the SVM models showed significantly higher performance than the RF models and NB models (*p* < 0.01, *p* = 0.047, respectively). Table B in the supplementary document summarizes the mean AUC results for all feature selection algorithms and machine learning models and for each imaging modality.

The mean AUCs of the models with the highest performance for each of the models using PET image features and CT image features are summarized in Table 3. Statistically significant differences in mean AUC were observed between all PET and CT feature models shown in Table 3. In all models for predicting each endpoint, the highest mean AUC was observed in the model combining PET features with SVM and Chi‐square test algorithm for predicting LR. For the prediction of LR, RM, RM‐RD, and LD‐RM‐DM, significantly higher mean AUCs were observed in models combining PET image features with SVM, NB, SVM, and SVM, respectively, than in models using CT image features. For the prediction of DM, LR‐RM, and LR‐DM, significantly higher mean AUC was observed in the model combining CT image features with SVM, NB, and SVM, respectively, than in models using PET image features. The specific hyperparameters and configurations used to develop each model shown in Table 3 are provided in the supplementary Document Table C.

**TABLE 3:** Maximum mean AUC of each model using PET image feature and CT image feature in predicting each endpoint.

	Mean AUC		
Recurrence pattern	CT features	PET features	*p*‐value
Local recurrence (LR)	0.575	0.646	**<0.01**
Regional lymph node metastases (RM)	0.573	0.611	**<0.01**
Distant metastases (DM)	0.645	0.602	**<0.01**
LR‐RM	0.559	0.552	**<0.05**
LR‐DM	0.597	0.585	**<0.01**
RM‐DM	0.557	0.587	**<0.01**
LR‐RM‐DM	0.549	0.574	**<0.01**

**FIGURE 3:**
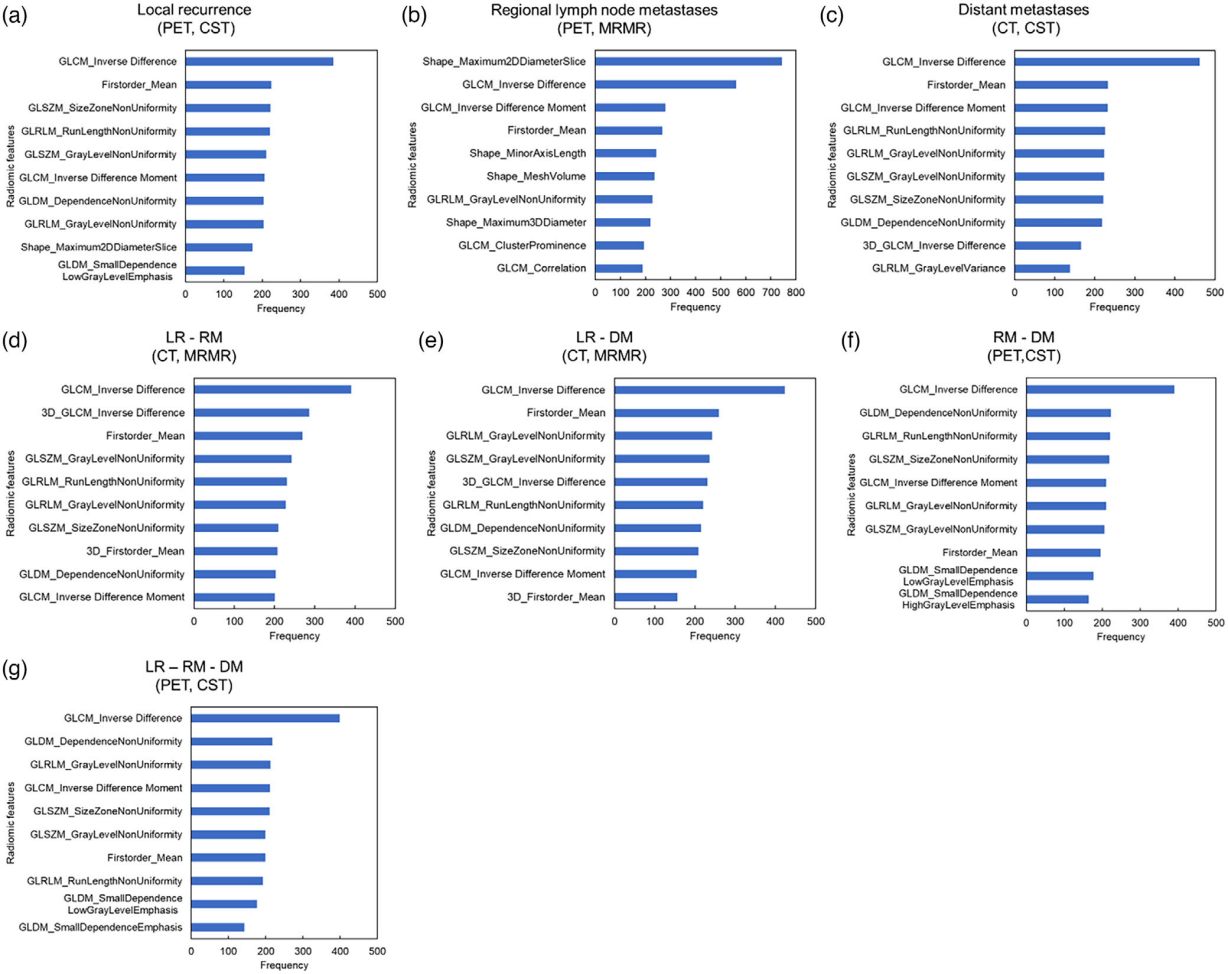
Histogram of the radiomic features that were selected within 1000 iterations to generate the model observed the highest AUC for each endpoint. The vertical axis shows the radiomic feature name, and the horizontal axis shows the number of times selected in 1000 iterations: (a) Local recurrence (LR), (b) Regional lymph node metastases (RM), (c) Distant metastases (DM), (d) LR‐RM, (e) LR‐DM, (f) RM‐DM, (g) LR‐RM‐DM.

The radiomic features that frequently appeared in the development of the predictive model for each endpoint at 1000 iterations of 0.632+ bootstrap were shown in Figure 3. The most frequently used features in the prediction model for the LR, DM, LR‐RM, LR‐DM, RM‐DM, and LR‐RM‐DM endpoints were the Inverse Difference, a Gray Level Co‐occurrence Matrix (GLCM) feature. The Inverse difference was selected 385, 462, 390, 423, 391, and 399 times during 1000 iterations, to develop predictive models for LR, DM, LR‐RM, LR‐DM, RM‐DM, and LR‐RM‐DM, respectively. In the model for predicting RM, the Maximum 2D diameter slice, the Shape feature, was selected 743 times during 1000 iterations and used to develop the model.

## 4 DISCUSSION

To the best of our knowledge, this is the first study to comprehensively evaluate the performance of machine learning models using CT and PET image features in predicting recurrence following SBRT in NSCLC patients. Similar previous research had predicted both local and distant recurrence by using data sets from patients treated with different prescribed doses and fractions respectively.^32^ Our AUC results demonstrated the potential of the radiomics model using PET image features in predicting local and regional recurrence (AUC = 0.646, 0.611), while the potential of the radiomics model using CT image features in the pretreatment prediction of distant recurrence was demonstrated (AUC = 0.645). In a previous report, a retrospective study of patients with early‐stage NSCLC after SBRT showed a possible improvement in overall survival in NSCLC patients who received adjuvant chemotherapy compared to those who received SBRT alone.^49^, ^50^ The model in this study shows the potential to predict the presence and type of recurrence using medical images acquired prior to treatment and may be applied to a system to support individualized radiotherapy, such as the addition of adjuvant chemotherapy after radiotherapy for patients at high risk of recurrence.

In this study, the high frequently used features to develop prediction models were the inverse difference, one of the GLCM features, and the maximum 2D diameter slice, one of the shape features. The GLCM feature is a second‐order gray‐level histogram first proposed by Haralick et al.^51^ GLCM is a matrix that enumerates the frequency of specific pixel value pairs in different directions within a region of interest (ROI) and describes the heterogeneity of intensity within the ROI. Inverse Difference, also known as Homogeneity, serves as a feature that quantifies the local uniformity within a given image (Equation 1).

(1) $$Inverse\;Difference\;=\;\sum_{k\;=\;0}^{N_{g}-1}\frac{p_{x-y}\left(k\right)}{1+k}$$

The maximum 2D diameter slice quantifies the maximum diameter of ROI in axial plane. The shape feature describes the size and shape of the ROI and is calculated only for the mask image, without using image intensity.

A previous study evaluating the performance of CT image features using univariate and multivariate analyses for predicting local and non‐local recurrence has reported that higher performance was observed in predicting local recurrence than non‐local recurrence.^28^ Comparing the AUCs for predicting local recurrence (LR) and non‐local recurrence (RM‐DM) with the CT image feature model in this study, the highest mean AUC was observed for predicting local recurrence (mean AUC for LR = 0.646 vs. mean AUC for RM‐DM = 0.587). These results agree with previous reports (AUC for local recurrence endpoints = 0.83 vs. AUC for non‐local recurrence endpoints = 0.60). Previous studies evaluating the performance of CT image features and PET image features for predicting local recurrence have reported that better predictive performance was observed in model using PET image features than the model using CT image features.^20^ Comparing the mean AUC of the prediction model developed using CT image features and the prediction model developed using PET image features for predicting local recurrence, we have found that the higher mean AUC was observed in model using PET image features than model using CT image features. These results agree with previous reports. Previous reports that predicted the risk of recurrence using models combining PET image features and machine learning algorithms showed that the highest predictive ability was observed in models using Random Forest classifiers. However, the results of our study differ in that the highest AUC was observed in the model using SVM for predicting recurrence except for RM and the highest AUC was observed in the model using NB for predicting RM.^27^ This difference in results may result from the use of pre‐treatment images of stage I–III patients who underwent curative resection in the dataset of the previous study, whereas pre‐treatment images of stage I–II patients who underwent SBRT were used in this study.

Our study has several limitations. First, the overall sample size was small (82 cases) and the number of recurrences relative to that sample size was also small. However, previous studies have reported analysis with similar sample sizes of 87 cases, and the 0.632+ bootstrap method used to evaluate model performance in this study has been shown to reduce the effect of bias in limited data sets.^20^, ^48^ The 82 cases data used in this study were divided into approximately 52 training data and 30 test data to assess performance. The occurrence rate of each recurrence pattern within the 82 cases varied, ranging from 18.29% to 47.56%. This variability might affect the generalization performance of the model. Second, the data set for this study consisted of 82 cases from a single institution. Therefore, the results of this study might be affected by variations in treatment strategies for the patients in the data set used and by differences in the devices employed for treatment. Moreover, only the internal validation was conducted to evaluate the performance of predictive model in this study. To obtain more comprehensive findings and to evaluate the generalizability and robustness of the predictive model developed in this study, further external validation studies using data sourced from multiple centers are needed. Third, the follow‐up period varied from patient to patient. In the data set of this study, the median follow‐up period was 38 months, with a range of 6–132 months. Some patients had shorter observation periods (10 patients were followed for less than 1 year) and thus could experience a recurrence later, even if they were expected to be recurrence‐free, which could decrease the performance of our model. Fourth, all PET images used in this study were acquired under free‐breathing conditions. Radiomic features extracted from free‐breathing PET images may affect the robustness of image features compared to gated PET images.^52^ Additionally, tumor segmentation of PET images was also performed using semi‐automatic segmentation in this study, which is available in open‐source software (3D‐Slicer). The reproducibility and reliability of this method have already been evaluated in previous studies.^29^, ^53^, ^54^ Finally, tumor segmentation in each CT image used GTV contours were drawn by a single expert for the purpose of SBRT treatment planning, so inter‐observer feature variability in the same CT image could not be assessed for CT features.^55^ However, some studies have reported that observer differences have little effect on the radiomic features for tumor segmentation in NSCLC patients on CT images.^56^

## 5 CONCLUSION

For the prediction of local, regional, and distant recurrence after SBRT, we developed and comprehensively evaluated recurrence prediction models based on radiomic features and machine learning of PET and CT images. Our results suggested that the model combining PET imaging features and SVM would be useful in predicting local and regional lymph node recurrence, and the model combining CT imaging features and SVM would be useful in predicting distant recurrence. Further prospective validation studies are needed to confirm the usefulness of the radiomic features.

## AUTHOR CONTRIBUTIONS

Conceptualization: Hikaru Nemoto, Masahide Saito, and Hiroshi Onishi. Data Curation: Hotaka Nonaka, Hiroaki Watanabe, Satoshi Funayama, and Yoko Satoh. Formal Analysis: Hikaru Nemoto, Masahide Saito, and Yoko Satoh. Methodology: Hikaru Nemoto, Masahide Saito, Yoko Satoh, and Hiroshi Onishi. Project Administration: Hikaru Nemoto, Masahide Saito, Takafumi Komiyama, and Hiroshi Onishi. Resources: Hikaru Nemoto, Takafumi Komiyama, Kan Marino, Shinichi Aoki, Hidekazu Suzuki, Naoki Sano, and Hiroshi Onishi. Software: Hikaru Nemoto and Masahide Saito. Supervision: Masahide Saito, and Hiroshi Onishi. Writing—Original Draft Preparation: Hikaru Nemoto, Masahide Saito, Yoko Satoh, and Hiroshi Onishi. Writing—Review & Editing: Hikaru Nemoto, Masahide Saito, Yoko Satoh, and Hiroshi Onishi.

## CONFLICTS OF INTEREST STATEMENT

Hikaru Nemoto has an endowed chair funded by Yamanashi PET Imaging Clinic and received a scholarship grant from GE Healthcare Pharma and Guerbet Japan KK. Hiroshi Onishi received research funds from Accuray, Elekta KK, and GE Healthcare Pharma, and patent royalties from Apex Medical. The remaining authors have no conflicts of interest to declare.

## Supporting information

Supporting Information

## Data Availability

The datasets generated and/or analyzed during the current study are not publicly available because the use of patietntdata (medical images and reports) other than by us is not approved by the patients, but are avilable from the corresponding author on reasonable request.
