# Supplementary material for: Evaluation of the performance of both machine learning models using PET and CT radiomics for predicting recurrence following lung stereotactic body radiation therapy: A single‐institutional study
Source: J Appl Clin Med Phys. 2024 Mar 4;25(7):e14322. doi: 10.1002/acm2.14322 (PMC11244675; doi:10.1002/acm2.14322)
Supplement: Supplementary file 1 — Supporting Information [file ACM2-25-e14322-s001.docx]

Supplemental document

Table A. The details of the radiomic features extracted using PyRadiomics in this study.

|  | **Radiomic feature name** | **Type** |
| --- | --- | --- |
| 1 | Energy | First order statistics |
| 2 | Total energy | First order statistics |
| 3 | Entropy | First order statistics |
| 4 | Minimum | First order statistics |
| 5 | 10th percentile | First order statistics |
| 6 | 90th percentile | First order statistics |
| 7 | Maximum | First order statistics |
| 8 | Mean | First order statistics |
| 9 | Median | First order statistics |
| 10 | Interquartile Range | First order statistics |
| 11 | Range | First order statistics |
| 12 | Mean Absolute Deviation (MAD) | First order statistics |
| 13 | Robust Mean Absolute Deviation (rMAD) | First order statistics |
| 14 | Root Mean Squared (RMS) | First order statistics |
| 15 | Standard Deviation | First order statistics |
| 16 | Skewness | First order statistics |
| 17 | Kurtosis | First order statistics |
| 18 | Variance | First order statistics |
| 19 | Uniformity | First order statistics |
| 20 | Mesh Volume | Shape-based (3D) |
| 21 | Voxel Volume | Shape-based (3D) |
| 22 | Surface Area | Shape-based (3D) |
| 23 | Surface Area to Volume ratio | Shape-based (3D) |
| 24 | Sphericity | Shape-based (3D) |
| 25 | Compactness 1 | Shape-based (3D) |
| 26 | Compactness 2 | Shape-based (3D) |
| 27 | Spherical Disproportion | Shape-based (3D) |
| 28 | Maximum 3D diameter | Shape-based (3D) |
| 29 | Maximum 2D diameter (Slice) | Shape-based (3D) |
| 30 | Maximum 2D diameter (Column) | Shape-based (3D) |
| 31 | Maximum 2D diameter (Row) | Shape-based (3D) |
| 32 | Major Axis Length | Shape-based (3D) |
| 33 | Minor Axis Length | Shape-based (3D) |
| 34 | Least Axis Length | Shape-based (3D) |
| 35 | Elongation | Shape-based (3D) |
| 36 | Flatness | Shape-based (3D) |
| 37 | Autocorrelation | Gray level co-occurrence matrix |
| 38 | Joint Average | Gray level co-occurrence matrix |
| 39 | Cluster Prominence | Gray level co-occurrence matrix |
| 40 | Cluster Shade | Gray level co-occurrence matrix |
| 41 | Cluster Tendency | Gray level co-occurrence matrix |
| 42 | Contrast | Gray level co-occurrence matrix |
| 43 | Correlation | Gray level co-occurrence matrix |
| 44 | Difference Average | Gray level co-occurrence matrix |
| 45 | Difference Entropy | Gray level co-occurrence matrix |
| 46 | Difference Variance | Gray level co-occurrence matrix |
| 47 | Joint Energy | Gray level co-occurrence matrix |
| 48 | Joint Entropy | Gray level co-occurrence matrix |
| 49 | Informational Measure of Correlation (IMC) 1 | Gray level co-occurrence matrix |
| 50 | Informational Measure of Correlation (IMC) 2 | Gray level co-occurrence matrix |
| 51 | Inverse Difference Moment (IDM) | Gray level co-occurrence matrix |
| 52 | Maximal Correlation Coefficient (MCC) | Gray level co-occurrence matrix |
| 53 | Inverse Difference Moment Normalized (IDMN) | Gray level co-occurrence matrix |
| 54 | Inverse Difference (ID) | Gray level co-occurrence matrix |
| 55 | Inverse Difference Normalized (IDN) | Gray level co-occurrence matrix |
| 56 | Inverse Variance | Gray level co-occurrence matrix |
| 57 | Maximum Probability | Gray level co-occurrence matrix |
| 58 | Sum Average | Gray level co-occurrence matrix |
| 59 | Sum Entropy | Gray level co-occurrence matrix |
| 60 | Sum of Squares | Gray level co-occurrence matrix |
| 61 | Small Area Emphasis (SAE) | Gray level size zone matrix |
| 62 | Large Area Emphasis (LAE) | Gray level size zone matrix |
| 63 | Gray Level Non-Uniformity (GLN) | Gray level size zone matrix |
| 64 | Gray Level Non-Uniformity Normalized (GLNN) | Gray level size zone matrix |
| 65 | Size-Zone Non-Uniformity (SZN) | Gray level size zone matrix |
| 66 | Size-Zone Non-Uniformity Normalized (SZNN) | Gray level size zone matrix |
| 67 | Zone Percentage (ZP) | Gray level size zone matrix |
| 68 | Gray Level Variance (GLV) | Gray level size zone matrix |
| 69 | Zone Variance (ZV) | Gray level size zone matrix |
| 70 | Zone Entropy (ZE) | Gray level size zone matrix |
| 71 | Low Gray Level Zone Emphasis (LGLZE) | Gray level size zone matrix |
| 72 | High Gray Level Zone Emphasis (HGLZE) | Gray level size zone matrix |
| 73 | Small Area Low Gray Level Emphasis (SALGLE) | Gray level size zone matrix |
| 74 | Small Area High Gray Level Emphasis (SAHGLE) | Gray level size zone matrix |
| 75 | Large Area Low Gray Level Emphasis (LALGLE) | Gray level size zone matrix |
| 76 | Large Area High Gray Level Emphasis (LAHGLE) | Gray level size zone matrix |
| 77 | Short Run Emphasis (SRE) | Grey level Run Length matrix |
| 78 | Long Run Emphasis (LRE) | Grey level Run Length matrix |
| 79 | Gray Level Non-Uniformity (GLN) | Grey level Run Length matrix |
| 80 | Gray Level Non-Uniformity Normalized (GLNN) | Grey level Run Length matrix |
| 81 | Run Length Non-Uniformity (RLN) | Grey level Run Length matrix |
| 82 | Run Length Non-Uniformity Normalized (RLNN) | Grey level Run Length matrix |
| 83 | Run Percentage (RP) | Grey level Run Length matrix |
| 84 | Gray Level Variance (GLV) | Grey level Run Length matrix |
| 85 | Run Variance (RV) | Grey level Run Length matrix |
| 86 | Run Entropy (RE) | Grey level Run Length matrix |
| 87 | Low Gray Level Run Emphasis (LGLRE) | Grey level Run Length matrix |
| 88 | High Gray Level Run Emphasis (HGLRE) | Grey level Run Length matrix |
| 89 | Short Run Low Gray Level Emphasis (SRLGLE) | Grey level Run Length matrix |
| 90 | Short Run High Gray Level Emphasis (SRHGLE) | Grey level Run Length matrix |
| 91 | Long Run Low Gray Level Emphasis (LRLGLE) | Grey level Run Length matrix |
| 92 | Long Run High Gray Level Emphasis (LRHGLE) | Grey level Run Length matrix |
| 93 | Coarseness | Neighbouring gray tone difference matrix |
| 94 | Contrast | Neighbouring gray tone difference matrix |
| 95 | Busyness | Neighbouring gray tone difference matrix |
| 96 | Complexity | Neighbouring gray tone difference matrix |
| 97 | Strength | Neighbouring gray tone difference matrix |
| 98 | Small Dependence Emphasis (SDE) | Grey level dependence matrix |
| 99 | Large Dependence Emphasis (LDE) | Grey level dependence matrix |
| 100 | Gray Level Non-Uniformity (GLN) | Grey level dependence matrix |
| 101 | Dependence Non-Uniformity (DN) | Grey level dependence matrix |
| 102 | Dependence Non-Uniformity Normalized (DNN) | Grey level dependence matrix |
| 103 | Gray Level Variance (GLV) | Grey level dependence matrix |
| 104 | Dependence Variance (DV) | Grey level dependence matrix |
| 105 | Dependence Entropy (DE) | Grey level dependence matrix |
| 106 | Low Gray Level Emphasis (LGLE) | Grey level dependence matrix |
| 107 | High Gray Level Emphasis (HGLE) | Grey level dependence matrix |
| 108 | Small Dependence Low Gray Level Emphasis (SDLGLE) | Grey level dependence matrix |
| 109 | Small Dependence High Gray Level Emphasis (SDHGLE) | Grey level dependence matrix |
| 110 | Large Dependence Low Gray Level Emphasis (LDLGLE) | Grey level dependence matrix |
| 111 | Large Dependence High Gray Level Emphasis (LDHGLE) | Grey level dependence matrix |

Table B.

Table C. Details of the parameters used to develop the prediction model that resulted in the highest observed AUC for each endpoint.

LR: Local recurrence

RM: Regional lymph node metastases

DM: Distant metastases

RLF: ReliefF algorithm

CST: Chi-square test algorithm

MRMR: Minimum redundancy maximum relevance algorithm

SVM: Support vector machine

RF: Random forest

NB: Naiive bayes

KNN: k-nearest neghborhood

rbf: Radial basis function
